# Social Media and Mental Health Among Young Adults in Cameroon

**DOI:** 10.34172/hpp.025.43746

**Published:** 2025-07-15

**Authors:** Jude Tsafack Zefack, Mbonjo Bitsie Dora, Brenda Mbouamba Yankam, Esua Alphonsius Fotindong, Vanessa Nkume, Nyeke James Tony, Ashu Martha Agbornyenty, Suh Colette Manka, Zita Wonjick Epse Khan Awa, Rose Nwenyoh Mbotoako, Abigail N. N Abamukong, Sally Tabe Njoh

**Affiliations:** ^1^Social Epidemiology Lab, Wuppertal, Germany; ^2^Department of Global Health and Bioethics, Euclid University, Bangui, Central African Republic; ^3^Break Sickle Cycle Association, Douala, Cameroon; ^4^Faculty of Mathematics, Ruhr-University Bochum, Bochum, Germany; ^5^Technical University of Applied Sciences Würzburg-Schweinfurt, Wurzburg, Germany; ^6^Faculty of Health Sciences, University of Buea, Buea, Cameroon; ^7^Medical Research Council Unit The Gambia at the London School of Hygiene & Tropical Medicine, Fajara, The Gambia; ^8^For Mom and Baby Foundation, Buea, Cameroon; ^9^Faculty of Medicine and Pharmaceutical Sciences, University of Douala, Douala, Cameroon; ^10^University of Exeter, Exeter, England; ^11^HappyMe Health Association, Buea, Cameroon

**Keywords:** Cameroon, Mental health, Social media, Young adults

## Abstract

**Background:**

Social media has become integral to daily life among young adults, offering opportunities for connection and self-expression and raising concerns about its impact on mental health. While global studies have examined these effects, evidence from Cameroon remains limited. This study assessed the relationship between social media use and psychological well-being among young Cameroonian adults.

**Methods:**

A cross-sectional online survey was conducted via Google Forms between September 2023 and April 2024 among Cameroonians aged 18-35. The questionnaire collected sociodemographic information, social media usage patterns, and self-reported mental health indicators. Descriptive statistics summarized platform use and engagement, while linear regression analysis examined associations between time spent on social media and mental health outcomes.

**Results:**

Among 151 respondents, WhatsApp (97.7%, 95% CI: 93.7-99.5%), Facebook (66.9%, 95% CI: 58.3-74.7%), and TikTok (43.4%, 95% CI: 34.9-52.1%) were the most used platforms. More respondents (39.74%) reported using social media for over five hours daily. Greater daily usage was linked with poorer self-reported mental health scores. Over one-third (37.1%) reported feelings of inadequacy related to social media, and 21.9% perceived a decline in their mental health since using it. Regression analysis indicated that prolonged social media use and cultural factors were associated with poorer self-rated mental health, while other sociodemographic factors were not significant predictors.

**Conclusion:**

Social media is deeply embedded in the lives of young Cameroonians, but it may negatively impact their mental well-being when usage is excessive. These findings highlight the urgent need for culturally tailored mental health interventions, digital literacy programs, and policies promoting healthier online engagement among youth.

## Introduction

 Nowadays, most young people utilize digital gadgets and social media. Approximately 95% of teenagers between the ages of 13 and 17 now have access to a smartphone, and at least 85% use social media.^1^ These trends extend to young adults, with approximately 96% of individuals aged 18–29 reporting smartphone ownership and 90% actively using social media.^1^ Given its widespread use, social media has become essential to young people’s everyday lives and interpersonal interactions.^2,3^ This digital context often enhances the conventional physical surroundings of development.^4,5^ Developing and maintaining friendships,^3,5^ negotiating autonomy,^3,6^ and projecting oneself to the public.^7,8^ These are among the developmental tasks adolescents and young adults typically engage in through social media.

 Youth development in the digital age has similarities and differences from the pre-social media era. New technologies are fulfilling comparable developmental demands.^9,10^ There is a considerable overlap or consistency between young people’s online and offline lives and activities.^4,5^ However, new features, possibilities, and difficulties for youth development have been brought about by the accessibility of social media. For example, youths may now communicate with their peers more often and instantly, which improves the flow of social support and raises expectations and demands for relationship maintenance.^11,12^ Social media content’s public nature and editability enable young people to showcase a desired image to a broad audience, yet not all forms of self-presentation are helpful.^13,14^

 Additionally, social media makes rich social information easily accessible, yet using it might lead to upward social comparison and a decline in well-being.^15,16^ Researchers have been devoting resources to gaining a more profound knowledge of the relationship between social media use and the well-being of adolescents and emerging adults, given the significance of social media in youth development. More detailed assessments of the conditions driving positive, negative, and neutral connections have lately come into focus.^17^ These distinctions are crucial because, even though social media and well-being are sometimes presented as either positive or negative contexts, the truth is more nuanced than this oversimplified picture.^18^ According to Yang et al,^1^ The main factors influencing the psychological effects of social media use are communication partners, activities, and reasons.^1^

 Several scholars have noted the unique consequences associated with various social media behaviors despite differences in the classifications used by the scholars.^1^ Most researchers acknowledged the distinctiveness of passive social media usage (such as surfing/browsing) but disagreed over categorizing active use. The three-activity typology proposed by Yang^19^ and Burke et al is comparable.^20^ Interactions aimed at a particular communication partner are referred to as interactive usage or directed communication (e.g., messaging/texting, commenting, “liking,” tagging). When social media content is created or published to reach a wider audience (e.g., by updating one’s status or publishing notes), it is considered active usage or broadcasting. However, this type of activity is only sometimes interactive. Because the material is consumed rather than created, passive use/content consumption (such as reading newsfeeds or perusing other people’s or one’s portfolios) is considered passive.^1^ Comparatively, passive use (i.e., consuming Information without direct interaction) is defined as activities facilitating direct interactions with others.^21,22^ This includes interactive use/directed communication and active use/broadcasting.

 Furthermore, to strengthen the theoretical underpinnings of this research, we integrate Bronfenbrenner’s ecological systems theory, which posits that a person’s mental health is influenced by various environmental factors, such as peers, family, cultural norms, and online interactions.^23^ Also, the biopsychosocial model provides an integrated approach, taking into account social influences (like peer comparison and cyberbullying), psychological processes (like anxiety and self-esteem), and biological predispositions.^24^ These models offer a more thorough framework for comprehending the impact of social media on Cameroonian young adults. We offer recommendations unique to Cameroon in addition to current policies and interventions. Digital literacy initiatives should be incorporated into school curricula to promote mental health awareness and critical social media engagement. Community-based programs can use conventional healing methods and nearby support systems to lessen stigma. Finally, government regulations and culturally sensitive content moderation must address ethical issues like data privacy and cyberbullying.

###  The Present Study

 The rapid growth of social media in Cameroon has raised concerns about its potential adverse effects on mental health. Users are exposed to stressors such as cyberbullying, social comparison, and information overload despite the increased connectivity. This study attempts to comprehend how social media use and mental health outcomes relate to one another in Cameroon, a nation with distinct sociocultural and economic characteristics. Cameroon’s collectivist culture emphasizes family and community, which could lead to social pressures on Facebook and WhatsApp. Economic differences also affect digital accessibility; cities are more likely to be online and aware of world trends, whereas people in rural areas might not have as much access. This emphasizes the need for studies to investigate how digital engagement affects mental health across various socioeconomic groups in Cameroon.

## Materials and Methods

###  Study Design and Sampling Frame

 This study employed a cross-sectional descriptive design to assess the relationship between social media use and mental health outcomes among Cameroonian youth. A non-probability convenience sampling strategy was used due to practical considerations, such as ease of access to participants and cost-effectiveness. The sampling frame consisted of Cameroonian young adults aged 18–35 years who were active on social media platforms and residing in Cameroon during the study period.

 Participants were recruited primarily through online distribution of a bilingual (English and French) Google Forms survey from September 2023 to April 2024. The survey link was disseminated widely via major social media platforms in Cameroon, including WhatsApp, Facebook, Instagram, and LinkedIn, using public pages, university and youth forums, and personal/professional networks. Eligibility criteria required that respondents be Cameroonian nationals aged 18–35, currently residing in Cameroon, and able to understand English or French. This approach was particularly suitable given the study’s focus on digitally connected youth and the widespread use of social media among this demographic.

 Although convenience sampling limits generalizability, it was appropriate for this exploratory research in a setting with limited national sampling frames for mental health research in youth populations. Recruitment efforts targeted various geographic regions and educational backgrounds to mitigate bias and enhance diversity.

###  Questionnaire

 The questionnaire was initially developed in English and subsequently translated into French using DeepL, an AI-powered translation tool known for its high linguistic accuracy.^25^ To enhance reliability, a back-translation procedure was implemented, whereby the French version was retranslated into English using the same tool and compared to the original to ensure semantic consistency and conceptual clarity. Although professional human translation was not employed, the instrument underwent critical review by three bilingual subject matter experts, who examined both versions to resolve ambiguities and enhance contextual accuracy.

 While the questionnaire was not formally pretested or subjected to statistical reliability testing, its content validity was strengthened through expert review and iterative refinement. The structured design and thorough evaluation contributed to the tool’s overall credibility and appropriateness for the Cameroonian context. However, future research must undertake formal pilot testing and psychometric validation to enhance the instrument’s rigor and generalizability.

 The final survey was administered in either English or French, based on participant preference, and comprised six sections: (1) sociodemographic information, (2) social media usage patterns, (3) mental health indicators, (4) social media activity types and perceived mental health effects, (5) moderating factors and (6) coping strategies related to negative experiences on social media.

###  Statistical Analysis

 All statistical analyses were conducted using R software, version 4.3.2. A significance level of *P* < 0.05 was used as the threshold for statistical significance. 95% confidence intervals (CIs) were calculated to indicate the precision of estimates.

 Mental health scores were computed using a 10-point Likert-type scale where participants rated their overall mental health from 1 (very poor) to 10 (excellent). No standardized clinical instruments were used; custom items developed for this study were utilized. Higher scores reflected better self-reported mental well-being.

 The dataset was checked for missing data. Imputation was not performed because of the low missingness rate (< 5%).

 A multiple linear regression analysis was employed to examine associations between mental health scores (dependent variable) and key independent variables, including gender, age, education level, region of residence, daily time spent on social media, cultural influences, social comparison, cyberbullying frequency, and social support level. These variables were selected based on theoretical relevance.

 Given the convenience sampling strategy via online platforms, clustering was not applied. Therefore, no design effect corrections were required. However, results should be interpreted cautiously, and future studies should use probability-based sampling for generalizability.

###  Ethical Considerations

 Formal ethical clearance was not sought, as the study involved minimal risk and collected no identifiable or sensitive personal data. However, ethical protocols were strictly followed per the Declaration of Helsinki. Participants were informed about the study’s purpose, confidentiality, and their right to withdraw via a pre-survey disclaimer. Consent was implied by completing the survey. Anonymity and data security were maintained throughout.

## Results

 A total of 151 respondents participated in the study, with females representing the majority (56.95%). Most participants were aged 21–25 years (40.39%), followed by those aged 18–20 years (23.84%) and 26–30 years (27.81%). The largest proportion resided in the Littoral region (56.29%), with smaller percentages from the Southwest (18.53%), Northwest (9.27%), Center (7.94%), West (4.63%), and other regions (3.31%). Regarding education level, nearly half held a bachelor’s degree (49.66%), while 23.17% had a master’s degree, 18.54% a PhD, 5.96% a high school diploma, and 2.64% other qualifications (Table 1). At the time of the study, 133 individuals, constituting 97.79% of the respondents, used WhatsApp. Other popular social media platforms used by respondents were Facebook (n = 91, 66.91%), TikTok (n = 59, 43.38%), Instagram (n = 54, 39.70%), Snapchat (n = 42, 30.88%), and LinkedIn (n = 40, 29.41%). And Twitter (n = 23, 16.91%) (Table 2). Similarly, most respondents spent over 5 hours per day on social media (n = 60, 39.73%), followed by 4 hours per day (n = 26, 17.21%) and 5 hours per day (n = 25, 16.55%) (Table 3). Furthermore, the frequency with which respondents post content on social media was as follows; “Occasionally” (n = 62, 41.05%), “Rarely” (n = 60, 39.73%), “Frequently” (n = 23, 15.23%), and “Very frequently” (n = 6, 3.97%) (Table 3). Also, most of our respondents (n = 112, 74.17%) did not engage in social comparison on social media. Some did engage in social comparison on social media (n = 17, 11.25%), and some needed clarification on whether they engaged (n = 22, 14.56%) (Table 3). Finally, when asked if they had experienced cyberbullying on social media, most respondents had “Never” (n = 71, 47.01%), followed by “Rarely” (n = 61, 40.39%), “Occasionally” (n = 18, 11.92%), and “Frequently” (n = 1, 0.66%) (Table 3).

**Table 1:** Sociodemographic characteristics of respondents (N = 151)

**Variables**	**Categories**	**N**	**%**
Gender	Female	86	56.95
	Male	65	43.05
Age (y)	18-20	36	23.84
	21-25	61	40.39
	26-30	42	27.81
	31-35	12	7.95
Region of residence	Littoral	85	56.29
	Southwest	28	18.53
	Northwest	14	9.27
	Center	12	7.94
	West	7	4.63
	Others	5	3.31
Education LEVEL	Bachelor’s degree	75	49.66
	Master’s degree	35	23.17
	PhD	28	18.54
	High school	9	5.9
	Others	4	2.64

*Note: *“Other” in *Region of residence* includes South (n = 2), Adamawa (n = 1), Far-North (n = 1), and Nord (n = 1). “Other” in *Education level* includes PharmD (n = 2), MD (n = 1), and Below High School (n = 1).

**Table 2:** Frequency of social media platform use among respondents

**Social media platform**	**N**	**%**	**95% CI**
WhatsApp	133	97.8%	93.7–99.5%
Facebook	91	66.9%	58.3–74.7%
TikTok	59	43.4%	34.9–52.1%
Instagram	54	39.7%	31.4–48.4%
Snapchat	42	30.9%	23.2–39.4%
LinkedIn	40	29.4%	21.9–37.8%
Twitter	23	16.9%	11.0–24.3%
YouTube	6	4.4%	1.6–9.4%
Telegram	3	2.2%	0.5–6.3%
Other	5	3.7%	1.2–8.4%

*Note: *“Other” combines all platforms with only one respondent each (Kingschat, Messenger, Pinterest, StarMaker, and Phoenix).

**Table 3:** Social media usage patterns, posting frequency, social comparison, and cyberbullying experiences among respondents (N = 151)

**Variable**	**Category**	**N**	**%**	**95% CI**
Daily time spent on SM (h)	Greater than 5	60	39.74%	31.9–48.0%
	5	25	16.56%	11.0–23.5%
	4	26	17.22%	11.6–24.2%
	3	18	11.92%	7.2–18.2%
	2	18	11.92%	7.2–18.2%
	1	1	0.66%	0.0–3.6%
	< 1	3	1.99%	0.4–5.7%
Post content on SM	Rarely	60	39.74%	31.9–48.0%
	Occasionally	62	41.06%	33.1–49.3%
	Frequently	23	15.23%	9.9–22.0%
	Very Frequently	6	3.97%	1.5–8.4%
Engaged in social comparison	No	112	74.17%	66.4–80.9%
	Yes	17	11.26%	6.7–17.4%
	Not sure	22	14.57%	9.4–21.2%
Experienced cyberbullying	Never	71	47.02%	38.9–55.3%
	Rarely	61	40.40%	32.5–48.7%
	Occasionally	18	11.92%	7.2–18.2%
	Frequently	1	0.66%	0.0–3.6%

###  Prevalence of Mental Health Issues

 The overall mental health status was scored from 1-10, 1 being very poor and 10 being excellent. 37 respondents (24.50%) reported a score of “10”, followed by 35 respondents (23.17%) with a score of “8”, 25 (16.55%) with a score of “9”, 19 (12.58%) with a score of “7” and 16 (10.59%) with a score of “5” (Table 4). In addition, when asked if they have ever been diagnosed with a mental health disorder, 143 (n = 94.70%) responded “No,” 4 (n = 2.64%) responded “Yes,” and 4 (n = 2.64%) were not sure (Table 5). Also, all those who had a previous mental health disorder sought professional help (n = 4, 100%) (Table 5). Furthermore, when asked if they ever felt anxious or stressed when using social media, 65 (43.04%) said: “Rarely,” followed by 42 (27.81%), “Never,” 37 (24.50%), “Occasionally,” and 7 (4.63%) “Frequently” (Table 5). Lastly, when asked if their mental health condition had improved or worsened since they started using social media, 76 (50.33%) reported “No change,” 42 (27.81%) reported “Improved,” and 33 (21.85%) reported “Worsened” (Table 5).

**Table 4:** Mental health scores among respondents

**Score**	**N**	**%**	**95% CI**
1	2	1.32%	0.2–4.7%
2	3	1.99%	0.4–5.7%
3	1	0.66%	0.0–3.6%
4	6	3.97%	1.5–8.4%
5	16	10.60%	6.2–16.6%
6	7	4.64%	1.9–9.3%
7	19	12.58%	7.7–19.0%
8	35	23.18%	16.7–30.7%
9	25	16.56%	11.0–23.5%
10	37	24.50%	17.9–32.2%

**Table 5:** Mental health diagnosis, professional help seeking, anxiety and stress levels, and perceived changes in mental health related to social media use (N = 151)

**Variables**	**Categories**	**N**	**%**
Previously diagnosed with a MH disorder	No	143	94
	Yes	4	2.64
	Not sure	4	2.64
Sought professional help in the past for MH concerns	No	139	92.05
	Yes	10	6.62
	Maybe	2	1.32
Felt anxious/stressed when using SM	Rarely	65	43.04
	Never	42	27.81
	Occasionally	37	24.50
	Frequently	7	4.63
Status of MH since started using SM	No change	76	50.33
	Improved	42	27.81
	Worsened	33	21.85

###  Impact of Social Media Activities on Mental Health

 When questioned on the most common social media activities, 122 (84.14%) engaged in “scrolling through the feed.” Other most common activities were “liking/reacting to post” (n = 80, 55.17%), “direct messaging” (n = 69, 47.59%), “posting status updates” (n = 68, 46.89%), “uploading photos/videos” (n = 67, 46.21%), and “commenting on post” (n = 53, 36.5%). Also, when asked if they have experienced feelings of inadequacy or low self-esteem because of social media activities, 55 (37.93%) responded “Yes,” 67 (46.21%) responded “No,” and 23 (15.86%) were not sure (Table 6). Furthermore, when asked if they felt a sense of belonging or exclusion when using social media, most respondents (n = 104, 68.87%) reported “Neither.” In comparison, 36 (23.84%) reported that they felt “Belonging,” and 11 (7.28%) reported that they thought “Excluded” (Table 6). When asked how social media usage affected their self-esteem, 88 (58.28%) reported a “Neutral,” 41 (27.15%) reported a “Positive impact,” and 22 (14.57%) reported a “Negative impact” (Table 6). Lastly, when asked about the Level of social support received through social media, 85 (56.29%) had “Moderate,” 59 (39.07%) reported “High,” and 7 (4.63%) “Low.”

**Table 6:** Impact of social media on self-esteem, sense of belonging, perceived social support, and feelings of inadequacy (N = 151)

**Variables**	**Categories**	**N**	**%**
Feelings of inadequacy due to SM activities	No	70	46.35
	Yes	56	37.08
	Not sure	25	16.55
Sense of belonging or exclusion when using SM	Neither	104	68.87
	Belonging	36	23.84
	Excluded	11	7.28
Impact of SM activities on self-esteem	Neutral	88	58.27
	Positive	41	27.15
	Negative	22	14.56
Level of social support received through SM	Moderate	85	56.29
	High	59	39.07
	Low	7	4.63

###  Moderating Factors and Coping Strategies Influencing the Relationship Between Social Media use and Mental Health

 The most reported offline activities known to moderate the impact of social media on mental health were “work or academic activities” (n = 105, 77.78%), Face-to-face interactions” (n = 98, 72.59%), and “hobbies and leisure activities” (n = 83, 61.48%). When asked to describe the level of social support received through social media, 85 (56.29%) responded with “Moderate” support, 59 (39.07%) responded with “Low” support, and 7 (4.63%) responded with “High” support (Table 7). In addition, when asked if cultural factors influenced how social media affects their mental health, 54 (35.76%) were “Not sure,” 51 (33.77%) reported that cultural factors did not influence how social media affects their mental health, and 46 (30.46%) reported that cultural factors influenced how social media affects their mental health (Table 7). Also, when asked if they were aware of any mental health resources provided on social media, 77 (50.99%) were “unaware,” 56 (37.08%) were “aware,” and 18 (11.92%) were “unsure” (Table 7). Furthermore, when asked about other strategies used to cope with stress or negative emotions related to social media use, 61 (48.41%) reported “hobbies and interest,” 48 (38.09%) reported “Educational engagements,” 46 (36.50%) reported “unfollowing or blocking,” and 42 (33.33%) reported “setting boundaries”.

**Table 7:** Social support, cultural influence, and awareness of mental health resources among respondents (N = 151)

**Variables**	**Categories**	**N**	**%**
Social support received through social media	Moderate	85	56.29
	Low	59	39.07
	High	7	4.63
Influence of cultural factors	Not sure	54	35.76
	No	51	33.77
	Yes	46	30.46
Aware of mental health resources provided on social media platforms	Unaware	77	50.99
	Aware	56	37.08
	Unsure	18	11.92

Table 8 presents the results of the multiple regression analysis examining predictors of mental health scores. After adjusting for sociodemographic characteristics, spending more time on social media daily was significantly associated with higher mental health scores, with all usage categories showing positive effects compared to the reference group (1 hour). Additionally, cultural factors were negatively associated with mental health scores, with both “Not sure” (95% CI: −1.99 to −0.24, *P* = 0.013) and “Yes” responses (95% CI: −1.90 to −0.02, *P* = 0.046) being statistically significant. Other variables, including gender, age, education level, region, social comparison, and cyberbullying frequency, did not show statistically significant associations.

**Table 8:** Multiple regression analysis predicting mental health status

**Variable**	**Category**	**95% CI**	***P*** ** value**
Gender	Female (Ref)	–	–
	Male	-0.54 to 1.04	0.527
Age	18–20 (Ref)	–	–
	21–25	-1.62 to 0.54	0.323
	26–30	-1.21 to 1.02	0.869
	31–35	-0.61 to 2.68	0.214
Education	Bachelor’s (Ref)	–	–
	Below high school	-3.47 to 5.11	0.706
	High school	-1.58 to 1.67	0.956
	Master’s degree	-0.89 to 1.16	0.800
	MD	-1.64 to 7.76	0.200
	PharmD	-3.65 to 2.59	0.737
	PhD	-0.33 to 1.95	0.163
Region of residence	Adamaoua (Ref)	–	–
	Centre	-4.90 to 4.25	0.889
	Far North	-7.20 to 5.26	0.758
	Littoral	-4.35 to 4.43	0.985
	Nord	-2.99 to 9.87	0.291
	Northwest	-3.66 to 5.49	0.691
	South	-3.95 to 6.62	0.618
	Southwest	-3.57 to 5.19	0.714
	West	-3.86 to 5.29	0.759
Hours spent on social media	1 hour (Ref)	–	–
	2 hours	3.14 to 12.50	0.0012 **
	3 hours	1.68 to 11.00	0.0082 **
	4 hours	1.84 to 11.10	0.0065 **
	5 hours	2.07 to 11.30	0.0049 **
	> 5 hours	1.71 to 10.90	0.0075 **
	< 1 hour	1.18 to 11.70	0.0169 *
Social comparison	No (Ref)	–	–
	Not sure	-1.78 to 0.45	0.237
	Yes	-0.97 to 1.43	0.706
Cyberbullying frequency	Frequently (Ref)	–	–
	Never	-1.24 to 7.94	0.151
	Occasionally	-1.93 to 7.58	0.242
	Rarely	-1.61 to 7.71	0.197
Social support level	High (Ref)	–	–
	Low	-1.34 to 2.42	0.573
	Moderate	-1.51 to 2.14	0.734
Cultural factors	No (Ref)	–	–
	Not sure	-1.99 to -0.24	0.0134 *
	Yes	-1.90 to -0.02	0.0465 *

Significance codes: * *P*< 0.05; ** *P* < 0.01. Reference categories: Female (Gender), 18–20 (Age), Bachelor’s (Education), Adamaoua (Region), 1 hour/day (Hours spent on social media), No (Social Comparison), Frequently (Cyberbullying Frequency), High (Social Support Level), No (Cultural Factors).

## Discussion

 This study examined the complex link between the use of social media and the mental health of young adults in Cameroon, providing insight into a range of sociodemographic traits, patterns of social media use, assessments of mental health, coping strategies, and moderating factors. The results provide a substantial contribution to the current discussion on the effects of social media on mental health, especially when considering a dynamic and varied group of people like young adults in Cameroon.

 The research participants’ sociodemographic profile showed a relatively balanced gender distribution, with the majority living in Cameroon’s Littoral area and falling between the ages of 21 and 30. The respondents had a very high level of education; the majority had at least a bachelor’s degree. This demographic profile illustrates how social media is becoming increasingly prevalent among educated young adults in Cameroon’s cities (Table 1).

 The study participants’ lives were increasingly integrated with social media; WhatsApp was the most popular app used by the most significant number of people, followed by Facebook, TikTok, and more (Table 2). These findings are supported by a recent article by the Pew Research Center, which reported that WhatsApp, Facebook, and TikTok dominate the social media landscape in middle-income nations.^26^ Young adults in Cameroon widely use social media; many spend more than five hours daily on it. These usage patterns highlight the pervasiveness of these platforms in daily life. Users had a variety of engagement patterns, as seen by the frequency of content uploading, with a significant portion just sometimes or infrequently engaged (Table 3).

 Regarding mental health evaluation, the findings indicate that 21.85% of participants reported worsened mental health since using social media (Table 5). Social comparison can have a particularly significant effect in Cameroon, where collectivist values are shared. Particularly for young adults juggling social expectations with personal goals, the pressure to maintain a positive online persona may contribute to stress. The need for culturally appropriate interventions that combine traditional and digital mental health support is further highlighted by the possibility that stigma around mental health in rural areas may discourage people from seeking help. This finding is similar to a previous study that found that university students’ use of social media was positively correlated with their psychological and social well-being through online social support and self-esteem.^27^

 Various social media activities existed, and scrolling through the feed was the most popular. Additionally, 37.93% of respondentsreported that their use of social media had caused them to feel inadequate or low in self-worth, underscoring the possible harm these sites may do to one’s sense of oneself. Similar findings were released by the Mental Health Foundation of Britain, indicating that millions of teenagers in the country worry about body image and blame social media for this issue.^28^ Most respondents did not have a strong sense of exclusion or belonging when using social media, suggesting a complex link between social media use and social connectivity (Table 6). Furthermore, hobbies, face-to-face interactions, and limiting social media use were reported as coping mechanisms. These strategies are consistent with meaning-focused coping frameworks,^29^ emphasizing adaptive methods for managing digital stressors. Socioeconomic limitations, however, might make it more challenging to access some coping strategies, like leisure activities. This emphasizes how policy interventions are needed to support offline engagement opportunities for young people from low-income families.

 We suggest the following interventions to address the identified mental health issues:

- Digital literacy programs: Schools should include social media education to assist young people in responsibly navigating online environments. Concerns about body image should be covered in these programs, especially the impact of Eurocentric beauty standards on sites like TikTok.
- Community-based mental health support: Given Cameroon’s insufficient mental health infrastructure, it is crucial to establish peer-led online support groups and culturally competent counseling services. Organizations like the LIFAFA Research Organisation are already making significant strides.^30^ To maximize impact, stakeholders should actively support and invest in expanding such initiatives nationwide, ensuring greater accessibility and sustainable mental health support for needy communities.
- Platform accountability measures: Localized content moderation tactics, such as addressing region-specific mental health issues and fighting cyberbullying in local languages, should be implemented by social media companies.
- Policy integration: These interventions will be more effective and long-lasting if they align with UNESCO’s digital inclusion frameworks and Cameroon’s National Mental Health Policy.

## Strengths and Limitations

 This study is the first to employ a bilingual questionnaire for both English and French speakers in Cameroon, guaranteeing inclusion and capturing a range of viewpoints on social media and mental health among young adults in Cameroon. The thoroughness with which the data was gathered, encompassing sociodemographic traits, social media usage habits, mental health evaluations, coping strategies, and moderating variables, enhanced the scope and richness of the results, offering significant perspectives for academics, mental health professionals, and policymakers. Also, the results could be representative because of the sample’s diversity, which includes individuals from different areas, educational levels, and social media usage habits.

 This study has potential limitations, including sampling bias due to social media recruitment, which may overrepresent digitally active individuals and underrepresent those with limited internet access. Also, it was conducted in English and French, excluding speakers of local languages; future research should include multilingual adaptations for broader representation. In addition, Self-report biases, such as social desirability, may have influenced the high percentage of respondents reporting “excellent” mental health. Additionally, the lack of validated mental health measures raises concerns about the accuracy of findings. Comparing results with studies using standardized tools like PHQ-9 and GAD-7 could help determine whether discrepancies arise from cultural differences or methodological limitations.

## Conclusion, Implications, and Future Directions

 This study delves into the complex relationship between social media use and the mental health of young adults in Cameroon, revealing how sociodemographic factors, cultural influences, and usage habits impact psychological well-being. The findings emphasize the need for a holistic and tailored approach to address mental health issues in this population, adding valuable insights to the growing field of digital mental health. The research lays the groundwork for developing evidence-based interventions specifically suited to Cameroonian youth’s needs by clarifying these dynamics. Future research should use validated instruments and larger samples to improve reliability. Longitudinal studies could reveal causal links between social media use and mental health over time, while qualitative methods like interviews can offer deeper insights into user experiences. Intervention studies are needed to test strategies for enhancing positive outcomes. Comparative studies across demographics could identify impact differences, and examining cultural factors could support culturally tailored mental health interventions.

## Competing Interests

 The authors declare that they have no competing interests.

## Ethical Approval

 This manuscript is the authors’ original work. All participants engaged in the research voluntarily and anonymously. Their data are stored in coded materials and databases without personal data.
